# Variation in COVID-19 Disease Severity and Clinical Outcomes Between Different ABO Blood Groups

**DOI:** 10.7759/cureus.21838

**Published:** 2022-02-02

**Authors:** Diyaa H Bokhary, Nidal H Bokhary, Lamees E Seadawi, Ahlam M Moafa, Hashim H Khairallah, Abdullah A Bakhsh

**Affiliations:** 1 Emergency Medicine, King Abdulaziz University Hospital, Jeddah, SAU; 2 Family Medicine, King Abdulaziz University Hospital, Jeddah, SAU; 3 Medicine, King Abdulaziz University Hospital, Jeddah, SAU

**Keywords:** abo blood group, outcome, severity, coronavirus, covid 19

## Abstract

The primary objective of this study was to explore whether coronavirus disease 2019 (COVID-19) severity and outcomes varied between different ABO blood groups. This retrospective study included 363 COVID-19 confirmed patients who had their blood group recorded in the hospital medical records, from March to June 2020. Data representing demographics, clinical features, vital signs, laboratory findings, and COVID-19 outcomes were collected. Multivariate logistic regression was used for analysis and the results were adjusted for sociodemographic, clinical, and laboratory variables. The patients’ mean age was 50 ± 17.8 years. Of the 363 patients, 30% were blood group A, 22.3% were blood group B, 8.8% were blood group AB, and 38.8% were blood group O. Bivariate analysis showed that patients with blood group AB were more likely to be free of any medical disease (65.6%) compared to other blood groups (p = 0.007). Fever was the most common presenting complaint (66.7%), and it did not significantly vary with changes in ABO blood groups (p = 0.230). Regarding laboratory characteristics, only C-reactive protein (CRP) levels were significantly associated with the blood groups, with high levels seen in blood groups A, B, and O (p = 0.036). In multivariate analysis, variations in emergency department (ED) disposition, requirement of intensive care unit care, and requirement of mechanical ventilation were not statistically significant among the different ABO blood groups. Furthermore, no correlation was found between hospital death and the different ABO blood groups. In conclusion, COVID-19 is most prevalent among patients with blood group O and least prevalent among those with blood group AB. No particular blood group had worse COVID-19 disease severity and outcomes than other blood groups. Therefore, we believe that ABO blood grouping should not be used as a major assessment tool for COVID-19 disease severity and outcome, and other known risk factors should be investigated.

## Introduction

In December 2019, the coronavirus disease 2019 (COVID-19) started to emerge in Wuhan, China, and rapidly spread across the world. By the 11th of March 2020, the WHO declared COVID-19 a global pandemic [1-2]. There has been a dramatic rise in the number of COVID-19 infected patients because of its rapid transmission [3]. To date, the number of COVID-19 confirmed patients is 276,436,619, and the mortality rate has reached 5,374,744 deaths [4].

The ABO blood group system is defined by the polymorphism of complex carbohydrate structures of glycoproteins and glycolipids expressed at the extracellular surface of erythrocytes or other cells, such as epithelium, sensory neurons, platelets, and vascular endothelium. The long arm of chromosome 9 contains the ABO gene, which determines the ABO blood type [5-6]. Previous observational studies have reported a relationship between ABO blood groups and certain defined pathologies. Dentali et al. confirmed the link between ABO blood groups and thrombosis risk in general, in which individuals with non-O blood groups were more likely to experience thrombotic episodes of venous or arterial origin [6]. Another study found an association between ABO blood groups and viral infections such as rotavirus, norovirus (NoV), dengue virus, Norwalk virus, and hepatitis B virus [7].

There are many significant factors that can play a major role in predicting COVID-19 outcomes, including sex, age, and clinical and laboratory findings [8-9]. In addition, comorbidities such as hypertension, diabetes mellitus, and asthma were among the strongest predictors of COVID-19 outcome. Furthermore, emerging studies have shown that ABO blood groups may also play a major role in the course of illness and in the outcome of COVID-19 patients; for example, COVID-19 was found to be less prevalent among people with blood group O. On the other hand, people with blood group A were more susceptible to acquiring COVID-19 with a higher mortality rate [9-11].

Moreover, Hoiland et al. stated that COVID-19 patients with blood groups A or AB are at a higher risk of requiring mechanical ventilation, continuous renal replacement therapy (CRRT), and prolonged intensive care unit (ICU) length of stay than other blood groups [10]. Additionally, the A allele of the ABO blood group is associated with an increased risk of developing cardiovascular disease in general [12]. Therefore, COVID-19 patients with A blood group, who are diagnosed with cardiovascular diseases, particularly hypertension, are more likely to develop more serious infections than others [12].

Multiple studies have confirmed the relationship between ABO blood groups and COVID-19 severity and outcomes. However, similar studies have not been conducted in the Kingdom of Saudi Arabia (KSA).

The main goal of this retrospective study was to evaluate the disease severity and clinical outcomes of COVID-19 patients in relation to different ABO blood groups.

## Materials and methods

This retrospective study was approved by the Institutional Ethical Review Board of King Abdulaziz University Hospital.

The study included all COVID-19 patients with polymerase chain reaction (PCR) confirmation test who had a recorded blood group in the system at King Abdulaziz University Hospital (KAUH), Kingdom of Saudi Arabia (KSA), Jeddah, from March to June 2020.

A total of 1039 COVID-19 patients were initially enrolled. Among them, 676 did not have a recorded ABO blood group and were, therefore, excluded from the study. Thus, 363 patients with a recorded ABO blood group were included in the final sample size.

We used a well-crafted data sheet composed of five main sections: demographics, clinical features, vital signs, laboratory findings, and COVID-19 outcomes. First, demographic information included age, sex, nationality, and comorbidities. Following this, clinical features, which included presenting symptoms to the emergency department (ED), were divided into two main categories: respiratory and non-respiratory symptoms. Initial ED vital signs and laboratory findings, included complete blood count (CBC), ABO blood group, renal function, liver function, cardiac enzymes, coagulation profile, lipid profile, and ferritin, C-reactive protein (CRP), and procalcitonin as inflammatory markers. Finally, disposition from the ED was categorized as either discharge from the hospital, admission to the isolation ward, or admission to the intensive care unit (ICU). Disease outcome was either recovery or death. The mechanical ventilation requirements for the admitted patients were also recorded.

Data were extracted from the medical records and saved on the laptop of the principal investigator in an electronic Google Form and Microsoft Excel file. Statistical analysis was performed using the Statistical Package for the Social Sciences version 23. Initially, continuous numerical variables, such as CBC, were grouped into low, normal, and high readings, based on our hospital reference ranges. The skewness and kurtosis scores were calculated to test the normality of our data. Frequency and mean were used as applicable in the univariate analysis, whereas the Chi-square test and analysis of variance (ANOVA) test were used as applicable in bivariate analysis to measure the association between each dependent variable and ABO blood groups. Finally, a logistic regression test was conducted as a multivariate analysis between the different COVID-19 outcomes and ABO blood groups. The results of the multivariate analysis were adjusted for all sociodemographic, clinical, and laboratory variables. In bivariate and multivariate analyses, a p-value of less than 0.05 was considered as significant.

## Results

The primary objective of our study was to explore whether COVID-19 disease severity and outcomes vary between different ABO blood groups.

Based on our inclusion criteria, 363 COVID-19 patients with a mean age of 50 ± 17.8 years were evaluated in the study. A majority of 283 (78%) patients were non-Saudi, and 206 (56.7%) were males. Most patients presented during May and June, with 177 (48.8%) and 162 (44.6%) patients, respectively. Of the 363 patients, 109 (30%) had blood group A, 81 (22.3%) had blood group B, 32 (8.8%) had blood group AB, and 141(38.8%) had blood group O. Among them, only 47 (13%) were in stable condition and discharged from the ED, while 316 (87%) required hospital admission. There were no significant associations between the sociodemographic characteristics and ABO blood groups. The sociodemographic characteristics of the participants are listed in Table 1.

**Table 1:** Distribution of sociodemographic characteristics among all COVID-19 patients.

		All n = 363 (100%)	A n = 109 (30%)	B n = 81 (22.3%)	AB n = 32 (8.8%)	O n = 141 (38.8%)	p value
Age (years)		50 ± 17.8	50 ± 15.4	51.4 ± 18	46 ± 16.7	50 ± 19.5	0.551
Gender:	Male	206(56.7%)	59(54.1%)	45(55.6%)	21(65.6%)	81(57.4%)	0.704
	Female	157(43.3%)	50(45.9%)	36(44.4%)	11(34.4%)	60(42,6%)	
Nationality:	Saudi	80(22%)	22(20.2%)	13(16%)	6(18.7%)	39(27.7%)	0.195
	Non-Saudi	283(78%)	87(79.8%)	68(84%)	26(81.3%)	102(72.3%)	
Month of presentation:	March	2(0.6%)	0(0%)	1(1.2%)	0(0%)	1(0.7%)	0.065
	April	22(6.0%)	8(7.3%)	6(7.4%)	0(0%)	8(5.7%)	
	May	177(48.8%)	57(52.3%)	49(60.5%)	13(40.6%)	58(41.1%)	
	June	162(44.6%)	44(40.4%)	25(30.9%)	19(59.4%)	74(52.5%)	
Emergency department disposition:	Discharge	47(13%)	12(11%)	13(16%)	3(9.4%)	19(13.5%)	0.693
	Admitted	316(87%)	97(89%)	68(84%)	29(90.6%)	122(86.5%)	

Patients’ medical history was assessed; 135 (37.2%) patients were not known to have any medical disease, which significantly varied among different ABO blood groups. For instance, patients with blood group AB were more likely to be without comorbidities (21/32, 65.6%) compared to other blood groups (p = 0.007). The most commonly reported condition was cardiovascular diseases in 165 patients (45.5%), which was correlated with the change in ABO blood groups [patients with cardiovascular condition who had blood group A (51.4%), blood group B (48.1%), blood group AB (15.6%), and blood group O (46.1%), p = 0.004]. Diabetes mellitus was the second most commonly reported condition in 134 patients (37%); however, it was not significantly variable among the different ABO blood groups. Other conditions were less frequently reported, such as renal disease, 39 (10.7%); cancer, 21 (5.8%); immunocompromised, 14 (3.9%); asthma, 11 (3%); and liver disease, 10 (2.7 %). There was no correlation between any of them and changes in ABO blood groups. The medical backgrounds of the different ABO blood groups are presented in Table 2.

**Table 2:** Distribution of past medical history among all COVID-19 patients. COPD, chronic obstructive pulmonary disease; DM, type 2 diabetes mellitus

	All n = 363 (100%)	A n = 109 (30%)	B n = 81 (22.3%)	AB n = 32 (8.8%)	O n = 141 (38.8%)	p value
Without comorbidities	135(37.2%)	37(33.9%)	29(35.8%)	21(65.6%)	48(34%)	0.007
Cardiovascular	165(45.5%)	56(51.4%)	39(48.1%)	5(15.6%)	65(46.1%)	0.004
DM	134(37%)	38(34.9%)	31(38.3%)	10(31.2%)	55(39%)	0.809
Cancer	21(5.8%)	8(7.3%)	3(3.7%)	0(0%)	10(7.1%)	0.316
Asthma	11(3%)	3(2.7%)	6(7.4%)	0(0%)	2(1.4%)	0.056
Immunocompromised	14(3.9%)	5(4.6%)	3(3.7%)	0(0%)	6(4.2%)	0.681
Renal disease	39(10.7%)	14(12.8%)	8(9.9%)	1(3.1%)	16(11.3%)	0.465
Liver disease	10(2.7%)	4(3.7%)	0(0%)	0(0%)	6(4.2%)	0.193
COPD	6(1.6%)	2(1.8%)	1(1.2%)	0(0%)	3(2.1%)	0.839

Fever was the most common presenting complaint, with a frequency of 242 (66.7%), followed by cough 202 (55.6%) and dyspnea 186 (51.2%). Other reported presenting symptoms in descending order of frequency were: nausea and vomiting, 48 (13.2%); abdominal pain, 37 (10.2%); diarrhea and sore throat, each 33 (9.1%). No significant association was found between the presenting symptoms and changes in ABO blood groups, except for cough, which was more likely seen in patients with blood group AB [patients with cough who had blood group A (59.6%), blood group B (56.8%), blood group AB (62.5%), and blood group O (50.3%), p = 0.033). Detailed information regarding the distribution of the presenting symptoms is presented in Table 3.

**Table 3:** Distribution of presenting symptoms among all COVID-19 patients.

	All n = 363 (100%)	A n = 109 (30%)	B n = 81 (22.3%)	AB n = 32 (8.8%)	O n = 141 (38.8%)	p value
Fever	242(66.7%)	68(62.4%)	56(69.1%)	26(81.2%)	92(65.2%)	0.230
Cough	202(55.6%)	65(59.6%)	46(56.8%)	20(62.5%)	71(50.3%)	0.033
Runny nose	24(6.6%)	7(6.4%)	8(9.9%)	1(3.1%)	8(5.7%)	0.051
Dyspnea	186(51.2%)	62(56.9%)	43(53.1%)	17(53.1%)	64(45.4%)	0.324
Chest pain	24(6.6%)	8(7.3%)	2(2.4%)	0(0%)	14(9.9%)	0.068
Nausea and vomiting	48(13.2%)	16(14.7%)	13(16%)	4(12.5%)	15(10.6%)	0.659
Abdominal pain	37(10.2%)	15(13.8%)	3(3.7%)	3(9.4%)	16(11.3%)	0.140
Diarrhea	33(9.1%)	6(5.5%)	11(13.6%)	5(15.6%)	11(7.8%)	0.132
Headache	22(6.1%)	7(6.4%)	8(9.9%)	2(6.2%)	5(3.5%)	0.300
Weakness	4(1.1%)	2(1.8%)	2(2.4%)	0(0%)	0(0%)	0.278
Fatigue	22(6.6%)	6(5.5%)	6(7.4%)	2(6.2%)	8(5.7%)	0.949
Bodyache	14(3.8%)	5(4.6%)	3(3.7%)	2(6.2%)	4(2.8%)	0.789
Sore throat	33(9.1%)	10(9.2%)	9(11.1%)	4(12.5%)	10(7.1%)	0.675

Regarding laboratory characteristics, 217 (59.8%) had a normal white blood cell (WBC) count and 264 (72.7%) had a normal platelet count. Most of our samples had a normal renal function, urea, and creatinine, with 201 (55.3%) and 225 (62%) patients, respectively. Generally, inflammatory markers were elevated; for example, 217 (59.8%) had high D-dimer levels, 177 (48.8%) had high ferritin levels, 290 (79.9%) had high C-reactive protein (CRP) levels, and 183 (50.4%) had high lactate dehydrogenase (LDH) levels. Among all reported laboratory characteristics, we found that only CRP levels were significantly correlated with different ABO blood groups, in which high CRP levels were observed mainly among patients with blood groups A, B, and O [80% of patients with blood group A, 82.7% of patients with blood group B, 71.9% of patients with blood group AB, and 80.1% of patients with blood group O had elevated CRP levels (p = 0.036)]. Table 4 lists the detailed distribution of the laboratory characteristics among the different ABO blood groups.

**Table 4:** Distribution of laboratory characteristics among all COVID-19 patients. WBC, white blood count; INR, international normalized ratio; CRP, C-reactive protein; LDH, lactate dehydrogenase

Characteristics		All n = 363 (100%)	A n = 109 (30%)	B n = 81 (22.3%)	AB n = 32 (8.8%)	O n = 141 (38.8%)	p value
WBC count (K\uL)	Low (<4.5)	60(16.5%)	21(19.3%)	12(14.8%)	4(12.5%)	23(16.3%)	0.471
	Normal (4.5 -11.5)	217(59.8%)	68(62.4%)	41(50.6%)	20(62.5%)	88(62.4%)	
	High (>11.5)	72(19.8%)	16(14.7%)	23(28.4%)	7(21.9%)	26(18.4%)	
Hemoglobin (g\dL)	Low (<12)	165(45.4%)	52(47.7%)	34(42%)	7(21.9%)	72(51%)	0.095
	Normal (12 - 16)	176(48.4%)	51(46.8%)	41(50.6%)	24(75%)	60(42.5%)	
	High (>16)	8(2.2%)	2(1.8%)	1(1.2%)	0(0%)	5(3.5%)	
Platelet count (K\uL)	Low (<150)	68(18.7%)	24(22%)	13(16%)	2(6.2%)	29(20.6%)	0.584
	Normal (150 - 450)	264(72.7%)	78(71.5%)	58(71.6%)	27(84.4%)	101(71.6%)	
	High (>450)	17(4.7%)	3(2.7%)	5(6.1%)	2(6.2%)	7(5%)	
Urea (mmol\L)	Normal (2.5 - 6.4)	201(55.3%)	62(56.9%)	44(54.3%)	18(56.2%)	77(54.6%)	0.793
	High (>6.4)	146(40.2%)	44(40.4%)	31(38.3%)	12(37.5%)	59(41.8%)	
Creatinine (umol\L)	Normal (53 - 115)	225(62%)	64(58.7%)	51(63%)	21(65.6%)	89(63.1%)	0.600
	High (>115)	122(33.6%)	42(38.5%)	24(29.6%)	9(28.1%)	47(33.3%)	
INR (ratio)	Normal (0.85 - 1.3)	254(70%)	71(65.1%)	55(67.9%)	25(78.1%)	103(73%)	0.681
	High (>1.3)	66(18.2%)	21(19.3%)	16(19.7%)	4(12.5%)	25(17.7%)	
Troponin (ug\L)	Normal (0.02 - 0.04)	213(58.7%)	61(56%)	44(54.3%)	23(71.9%)	85(60.3%)	0.268
	High (>0.04)	84(23.1%)	27(24.8%)	16(19.7%)	5(15.6%)	36(25.5%)	
D-dimer (mg\L)	Normal (≤0.5)	80(22%)	27(24.8%)	20(24.7%)	11(34.4%)	22(15.6%)	0.227
	High (>0.5)	217(59.8%)	61(56%)	46(56.8%)	18(56.2%)	92(65.2%)	
Ferritin (ng\mL)	Normal (20 - 250)	122(33.6%)	41(37.6%)	27(33.4%)	10(31.2%)	44(31.2%)	0.174
	High (>250)	177(48.8%)	52(47.7%)	45(55.6%)	12(37.5%)	68(48.2%)	
CRP (mg\L)	Normal (≤3)	3(0.8%)	0(0%)	4(4.9%)	0(0%)	0(0%)	0.036
	High (>3)	290(79.9%)	87(80%)	67(82.7%)	23(71.9%)	113(80.1%)	
Procalcitonin (ng\mL)	Normal (≤0.15)	120(33.1%)	41(37.6%)	27(33.4%)	12(37.5%)	40(28.4%)	0.551
	High (>0.15)	143(39.4%)	40(36.7%)	36(44.4%)	12(37.5%)	55(39%)	
LDH (U\L)	Normal (100 - 240)	62(17.1%)	21(19.3%)	11(13.6%)	2(6.2%)	28(19.8%)	0.187
	High (>240)	183(50.4%)	61(56%)	40(49.4%)	19(59.4%)	63(44.7%)	

Among the admitted 316 patients, 102 (32.3%) were in critical condition and required ICU admission, and 101 (32%) were in respiratory distress and required mechanical ventilation. Unfortunately, 109 (34.5%) patients did not recover from COVID-19 and died. None of these variables were significantly different among the different ABO blood groups in the bivariate analysis, as shown in Table 5.

**Table 5:** Distribution of different outcomes among hospital admitted COVID-19 patients.

		All n= 316 (100%)	A n= 97 (%)	B n= 68 (%)	AB n= 29 (%)	O n= 122 (%)	p value
Hospital admission:	Ward	214(67.7%)	66(68%)	41(60.3%)	21(72.4%)	86(70.5%)	0.486
	ICU	102(32.3%)	31(32%)	27(39.7%)	8(27.6%)	36(29.5%)	
Mechanical ventilation requirement:	Yes	101(32%)	30(30.9%)	27(39.7%)	7(24.1%)	37(30.3%)	0.409
	No	215(68%)	67(69.1%)	41(60.3%)	22(75.9%)	85(69.7%)	
Overall hospital disposition:	Discharge	207(65.5%)	68(70.2%)	40(58.8%)	21(72.4%)	79(64.7%)	0.392
	Death	109(34.5%)	29(29.8%)	28(41.2%)	8(27.6%)	44(35.3%)	

In the multivariate analysis, ED disposition (admission rather than discharge) was not associated with the change in ABO blood groups [blood group A (odds ratio, OR 2.490; 95% confidence interval, CI 0.809-7.670; p = 0.223), blood group B (OR 0.761; 95% CI 0.230-2.518; p = 0.655), blood group AB (OR 3.153; 95% CI 0.541-18.381; p = 0.202), and blood group O (reference)], as shown in Table 6.

**Table 6:** Distribution of multivariate logistic regression test: ED disposition (admission) among different blood groups. Results are adjusted for sociodemographic and comorbidities variables. ED, emergency department

Blood groups	p value	Odds ratio	95% CI
A	0.112	2.490	0.809 – 7.670
B	0.655	0.761	0.230 – 2.518
AB	0.202	3.153	0.541 – 18.381
O	Reference	Reference	Reference

In addition, the requirement of ICU admission was not correlated with the change in ABO blood groups [blood group A (OR, 1.014; 95% CI, 0.384-2.677; p = 0.978), blood group B (OR 0.955; 95% CI 0.322-2.833; p = 0.933), blood group AB (OR 0.744; 95% CI 0.147-3.769; p = 0.721), blood group O (reference) Table 7]. Moreover, no association was found with mechanical ventilation requirement among the different ABO blood groups [blood group A (OR 0.862; 95% CI 0.336-2.209; p = 0.757), blood group B (OR 0.907; 95% CI 0.312-2.635; p = 0.858), blood group AB (OR 0.884; 95% CI 0.187-4.172; p = 0.876), and blood group O (reference) Table 8]. Furthermore, no correlation was found between death and different ABO blood groups [blood group A (OR 0.994; 95% CI 0.402-2.459; p = 0.990), blood group B (OR 1.216; 95% CI 0.408-3.620; p = 0.726), blood group AB (OR 1.099; 95% CI 0.223-5.423; p = 0.908), and blood group O (reference) Table 9]. 

**Table 7:** Distribution of multivariate logistic regression test: hospital admission to ICU rather than ward among different blood groups. Results are adjusted for sociodemographic and comorbidities variables. ICU, intensive care unit

Blood groups	p value	Odds ratio	95% CI
A	0.978	1.014	0.384 – 2.677
B	0.933	0.955	0.322 – 2.833
AB	0.721	0.744	0.147 – 3.769
O	Reference	Reference	Reference

**Table 8:** Distribution of multivariate logistic regression test: mechanical ventilation requirement among different blood groups. Results are adjusted for sociodemographic and comorbidities variables.

Blood groups	p value	Odds ratio	95% CI
A	0.757	0.862	0.336 – 2.209
B	0.858	0.907	0.312 – 2.635
AB	0.876	0.884	0.187 – 4.172
O	Reference	Reference	Reference

**Table 9:** Distribution of multivariate logistic regression test: overall hospital disposition (death) among different blood groups. Results are adjusted for sociodemographic and comorbidities variables.

Blood groups	p value	Odds ratio	95% CI
A	0.990	0.994	0.402 – 2.459
B	0.726	1.216	0.408 – 3.620
AB	0.908	1.099	0.223 – 5.423
O	Reference	Reference	Reference

## Discussion

This study aimed to examine the variation in the severity and outcomes of COVID-19 infection in relation to different ABO blood groups.

It was seen that COVID-19 infection was more prevalent in males (56.7%), which is supported by previous studies [13-15]. This can be explained by the multiple influencing factors of the female gender, including sex chromosomes and hormones, which increase their innate immunity compared to males. Therefore, females have a higher resistance to different infectious agents and stronger immune responses to pathogens than males [16].

The mean age of the patients was 50 ± 17.8 years. In fact, older people are more likely to present to EDs and are admitted to hospitals than younger healthy people, usually because older patients require additional observation and monitoring for any complications of COVID-19, which are less frequent in younger healthy people [17]. Furthermore, most of our patients who presented to the ED were admitted to the hospital (87%), which is supported by the fact that they were older and more prone to developing respiratory and systemic complications. Additionally, due to their older age, most of our patients had chronic medical diseases (62.8%).

The ABO blood group is an important indicator of the general susceptibility to infections, and it has a reliable value that predicts the possibility of having chronic medical diseases and different types of cancers [18]. We found no significant association between ABO blood groups and sociodemographic characteristics among COVID-19 patients.

However, there was a significant association between ABO blood groups and chronic diseases, as COVID-19 patients with AB blood group were found to have fewer chronic diseases compared to other blood groups. This could be related to the fact that only a small number of our patients had an AB blood group, which represented only 8.8% of our sample size, and the majority were in the non-AB blood group (91.2%). The most commonly reported medical issue was cardiovascular diseases (45.5%), which varied between the ABO blood groups and showed the highest coincidence with blood group A (51.4%), as noted in a previous study [19]. Consistent with other studies, the non-O blood group had a higher incidence and risk of developing cardiovascular diseases, which is related to the presence of high levels of von Willebrand factor (VWF), an important risk factor, which has been found to be the lowest in patients with O blood group [18, 20].

The initial presenting symptoms could have a wide range of variations among patients according to multiple factors, including patient age, immune status, and severity of infection. The most common symptoms were fever (66.7%), cough (55.6%), and dyspnea (51.2%). There was a significant association between cough and ABO blood group, as it was more common in COVID-19 patients with blood group AB, which is consistent with the findings of a previous study [21]. Another study conducted by the European Society of Cardiology stated that non-O blood groups have higher angiotensin-converting enzyme (ACE) inhibitor-induced cough activity in patients with hypertension [12]. This is consistent with our results, as the lowest percentage of coughs was observed among the O blood groups.

Most of our patients had high levels of inflammatory markers, including D-dimer (59.8%), ferritin (48.8%), CRP (79.9%), and LDH (50.4%). Interestingly, we found that CRP level was the highest among COVID-19 patients with blood groups A (80%), B (82.7%), and O (80.1%), and the lowest percentage was among patients in blood group AB (71.9%) (p = 0.036). Previous studies have shown that the higher the CRP level, the more severe the clinical course of COVID-19 infection [22]. This fact is consistent with the results of our study, which showed that the AB blood group patients had the lowest CRP levels; and consequently, had the lowest ICU admissions (27.6%) compared to other blood groups. However, since patients in blood group B had the highest CRP levels, they also had the highest percentage of ICU admissions among other blood groups.

Regarding COVID-19 severity, we found no significant association between different blood groups and ED disposition, the requirement of mechanical ventilation, or ICU admission. Some published papers are in agreement with our results, such as a meta-analysis that negated the association between blood groups and severity [23]. Another meta-analysis showed that although some studies reported an association of blood groups with disease severity overall, there was no sign of an association between blood groups and the severity of COVID-19 [24]. Another study stated that although patients with non-O blood groups had a higher incidence of COVID-19 infection, blood groups were not associated with disease course and severity [25]. According to a paper published in Wuhan, there might be a minimal association of blood group A in females with disease severity, but no association was reported with the other groups. On the other hand, few studies disagree with our findings. A study by Dai stated that blood group O is associated with a less severe COVID-19 disease course, as there are less likely chances of cardiovascular complications during disease activity [12]. Additionally, a systematic review stated that patients with blood group O have protective characteristics against severe COVID-19 [26], and this was supported by another study [19]. We believe that these differences in the literature are not due to a direct cause-and-effect between COVID-19 and blood groups; rather, it is an indirect effect. For instance, patients with non-O blood groups have a higher chance of cardiovascular events, and; when combined with COVID-19 infection, it results in worsening of overall patient condition and not necessarily COVID-19 infection. This belief can be supported by the two meta-analyses which stated that when correction was made for blood groups associated with severe COVID-19 infection, no association was found.

In our study, we found no significant association between blood groups and mortality outcomes of COVID-19 infection, which can be supported by a meta-analysis reporting similar outcomes [23]. Other studies have reported no significant association between blood groups and COVID-19 outcomes [27-28]. However, few other studies are in conflict with our results. One study found that patients with blood group B had significantly higher mortality rates in COVID-19 [29]. Two other studies found that blood group A was significantly associated with death in COVID-19 patients [30]. We believe that this difference is due to the same reason mentioned previously. Furthermore, while some studies found an association between COVID-19 outcomes and blood groups, the blood groups evaluated varied between papers; this, we believe, negates the true association between blood groups and outcome and supports our claim that other factors were responsible for the outcomes. 

## Conclusions

In conclusion, COVID-19 infection is more prevalent among patients with blood group O, followed by those with blood groups A, B, and then AB. No particular blood group had worse COVID-19 disease severity and outcomes than other blood groups. Therefore, we believe that ABO blood grouping should not be used as a major assessment tool for COVID-19 disease severity and outcome, and instead, other known risk factors should be investigated.
